# Trends in Tuberculosis Case Notification and Treatment Success, Haiti, 2010–2015

**DOI:** 10.4269/ajtmh.16-0863

**Published:** 2017-10-18

**Authors:** Macarthur Charles, Milo Richard, Patrice Joseph, Margarette R. Bury, Georges Perrin, Frantz Jean Louis, David L. Fitter, Barbara J. Marston, Varough Deyde, Jacques Boncy, Willy Morose, Jean W. Pape, David W. Lowrance

**Affiliations:** 1 Centers for Disease Control and Prevention, Port-au-Prince, Haiti;; 2 Programme National de Lutte contre la Tuberculose, Ministère de la Santé Publique et de la Population, Port-au-Prince, Haiti;; 3 Les Centres GHESKIO, Port-au-Prince, Haiti;; 4 Pan American Health Organization/World Health Organization, Port-au-Prince, Haiti;; 5 Laboratoire National de Santé Publique, Ministère de la Santé Publique et de la Population, Port-au-Prince, Haiti

## Abstract

Since the 2010 earthquake, tuberculosis (TB) control has been a major priority for health sector response and recovery efforts in Haiti. The goal of this study was to analyze trends in TB case notification in Haiti from the aggregate data reported by the National TB Control Program to understand the effects of such efforts. A total of 95,745 TB patients were registered for treatment in Haiti between 2010 and 2015. Three regions, the West, Artibonite, and North departments accounted for 68% of the TB cases notified during the period. Patients in the 15–34 age groups represented 53% (50,560) of all cases. Case notification rates of all forms of TB increased from 142.7/100,000 in 2010 to 153.4 in 2015, peaking at 163.4 cases/100,000 in 2013. Case notification for smear-positive pulmonary TB increased from 85.5 cases/100,000 to 105.7 cases/100,000, whereas treatment success rates remained stable at 79–80% during the period. Active TB case finding efforts in high-risk communities and the introduction of new diagnostics have contributed to increasing TB case notification trends in Haiti from 2010 to 2015. Targeted interventions and novel strategies are being implemented to reach high-risk populations and underserved communities.

## INTRODUCTION

Tuberculosis (TB) is a major public health problem in Haiti; disease rates are the highest in the Western Hemisphere, and TB is among the leading causes of death.^1,2^ Between 2005 and 2010, the Haiti National TB Program (Program National de Lutte contre la Tuberculose [PNLT]) consistently reported approximately 14,000 cases each year, although the World Health Organization (WHO) estimates the number of incident TB cases to be between 22,000 and 27,000,^1^ indicating a potential gap of as many as 13,000 (48%) cases that go undiagnosed and untreated in the country.

The earthquake of January 12, 2010, killed an estimated 200,000 people and displaced more than 1.5 million, forcing this population to settle under crowded conditions in camps and shelters.^3^ In the wake of the catastrophe, strengthening disease surveillance systems and controlling the transmission of TB assumed a new sense of urgency.^4,5^ PNLT and its partners initially focused efforts and resources on case detection through active case finding in high-risk populations living in camps, tents, and slums and on improving TB diagnosis. Using cough as a screening tool, community health workers (CHWs) identified those suspected of having TB and systematically referred them to health facilities for evaluation. One study examining this approach documented TB incidence rates as high as 1,165 cases per 100,000 in a Port-au-Prince slum.^6^ Table 1 summarizes the major TB-related activities that were implemented or strengthened during the period. More sensitive diagnostic methods, such as fluorescent light-emitting diode (LED) microscopy and the GeneXpert platform (Cepheid, Sunnyvale, CA) for simultaneous detection of *Mycobacterium tuberculosis* and rifampin resistance (Xpert MTB/RIF), were also rolled out at major TB centers throughout the country to rapidly diagnose and treat patients. To improve treatment of all cases of TB, the national TB guidelines were revised to include the short-course treatment based on using fixed-dosed combination drugs, and TB infection control measures were strengthened across the highest volume TB facilities, whereas cough surveillance was scaled up widely within public health facilities.

**Table 1:** Summary of TB-related activities in Haiti during the 2010–2015 period

Interventions	Diagnosis	Treatment	Prevention
Active case finding in urban centers, camps, and slums	Implementation of LED fluorescent microscopy at high burden TB centers	Implementation of short-course fixed-dosed combination drug treatment under DOTS	TB infection control training and implementation of guidelines
Implementation of cough surveillance at all TB centers	Implementation of GeneXpert at departmental laboratories and high-burden TB centers	Revision of multidrug-resistant-TB guidelines treatment	Isoniazid for all children under 5 and for HIV-positive individuals
Implementation of contact tracing surveillance at selected TB centers	Expansion of EQA for microscopy and GeneXpert testing	Expansion of MDR-TB treatment capacity	Advocacy for TB
			Celebration of World TB day
			Enhance leadership and governance at the central level

DOTS = directly observed treatment short course; EQA = external quality assurance; LED = light-emitting diode; TB = tuberculosis.

We analyzed aggregate TB data from the PNLT’s surveillance system to retrospectively describe trends in TB case notification by category, geographic department, gender, and age group for the period 2010–2015. We aimed to use the surveillance data to inform further strengthening of TB control in Haiti, in accordance with WHO recommendations.^7^

## MATERIALS AND METHODS

### Study population and setting.

We used TB notification data collected and aggregated by the Haiti Ministry of Health’s National TB Program (PNLT) from 2010 to 2015. The population of the study included all new and retreatment TB cases diagnosed in Haiti during the study period.

Of the 907 health facilities across Haiti’s 10 administrative departments, 234 diagnose and treat patients with TB (Center of Diagnostic and Treatment [CDT]).^8^ An additional 30 health facilities, known as Center of Treatment (CT), administer treatment to patients who already have a confirmation of TB diagnosis from another facility. Some of the CDTs also rely on CHWs who are members of the community trained to recognize the signs and symptoms of TB. Using cough as a screening tool, CHWs identify those in the community suspected of having TB and refer them or accompany them to health facilities for further evaluation.

The Haiti TB surveillance system consists of several paper-based registries that are used for data collection at the CDT and CT level; those include TB case, laboratory, contact tracing, and respiratory symptomatic registries.^9^ Before 2011, PNLT relied primarily on a local nongovernmental organization partner, International Child Care (ICC), for national-level TB surveillance data. In 2011, PNLT established its own case-based database and gradually incorporated the ICC surveillance data into the present system. Aggregate data collected from the paper registries are reviewed and validated during quarterly departmental meetings. A recent evaluation of the PNLT surveillance system found it to be robust and strongly performing with a high degree of completeness of the reporting of detected cases.^10^

### TB diagnosis and treatment.

TB was diagnosed and treated according to PNLT guidelines across all CDT and CT in the country.^11^ Diagnostic sputum smear microscopy examination is performed either with light microscopy after Ziehl–Neelsen staining or with LED fluorescent microscopy after auramine staining. Microscopists routinely reserve 5–10% of slides for quality assurance purposes.^12^ In the past 5 years, there has been > 95% concordance between slides read by departmental microscopists and slide recheckers at the National Public Health Laboratory. Twenty GeneXpert (Cepheid Inc., Sunnyvale, CA) machines were obtained and deployed to facilities across the country to increase access to testing for eligible patients. Each of Haiti’s 10 geographic departments has at least one GeneXpert equipment. According to the 2015 PNLT guidelines, patients eligible for Xpert MTB/RIF testing include human immunodeficiency virus (HIV)-positive patients, children under 10 years old, prisoners, patients with positive sputum smears past the third month of treatment, and symptomatic contacts of patients with confirmed multidrug-resistant TB. All TB molecular tests, culture, drug susceptibility testing, and treatment are provided free of charge. Anti-TB drugs are not available outside of the PNLT network.

In 2010, PNLT adopted the short-course TB treatment as part of the directly observed treatment short course strategy for all patients. Routine TB treatment uses fixed-dose combination drugs and lasts 6 months. During the intensive phase of treatment, patients receive isoniazid, rifampin, ethambutol, and pyrazinamide for a duration of 2 months. The 4-month continuation phase of treatment includes rifampin and isoniazid. Definitions of treatment outcomes (cured, treatment completed, treatment success, treatment failed, defaulted, died, lost to follow-up, not evaluated) were according to PNLT guidelines, which are aligned with those of the WHO.

### TB case definitions.

Smear-positive pulmonary TB is defined as TB with at least two initial sputum smear microscopy examinations positive for acid-fast bacilli or one positive smear and a chest radiograph showing findings consistent with pulmonary TB. Smear-negative TB is diagnosed by a physician after at least three smear microscopy examinations have been negative in patients with signs and/or symptoms of TB who showed no improvement after a course of antibiotics. Extrapulmonary TB is diagnosed based on clinical signs and symptoms of extrapulmonary TB and either a positive culture or histopathologic evidence of TB. However, because of limited histopathologic capacity, most cases of extrapulmonary TB in Haiti are diagnosed on clinical grounds.

### Statistical analysis.

Trends in notified TB cases, case notification rates (CNRs) for 2010–2015 were analyzed and described. The annual population estimates were obtained from the Institut Haïtien de Statistique et d’Informatique (IHSI).^13^ CNRs were measured as the number of TB cases notified during a given year divided by the population estimate for that year. Annual trends in notified cases and CNRs were analyzed by geographic department, gender, and age group. National trends in treatment success rates were also analyzed. Simple linear least squares regression was used to detect trends in CNRs over time, the independent variable being the year and the dependent variable being case notification or CNR.

All statistical analyses were carried out in Microsoft Excel (Microsoft Corp., Redmond, WA). *P* values of < 0.05 were considered statistically significant. Maps were generated using the ArcGIS software package (Esri, Redlands, CA).

### Ethical considerations.

The Centers for Disease Control and Prevention and PNLT deemed this project to be a public health program evaluation and not to involve human subjects since it involved routine public health activities.

## RESULTS

### General characteristics of the study population.

**Figure 1.**
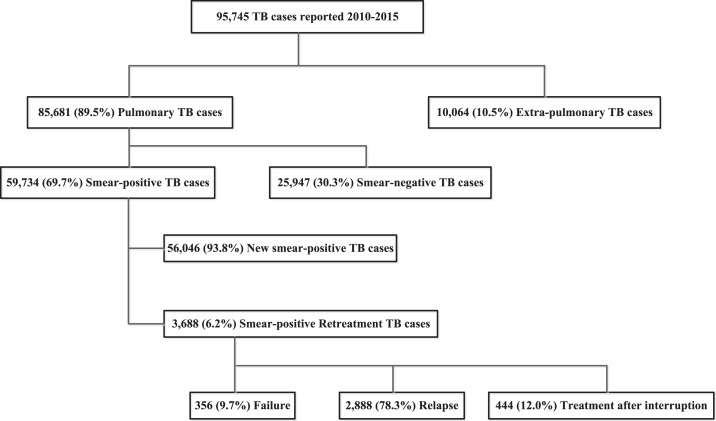
Categories of tuberculosis disease among notified cases, Haiti, 2010–2015.

From 2010 to 2015, a total of 95,745 cases of TB, including 90,840 (94.9%) new cases and 4,905 (5.1%) retreatment cases, were reported to PNLT (Figure 1). There were 85,681 (89.5%) pulmonary TB and 10,064 (10.5%) extrapulmonary TB cases. Among the pulmonary TB cases, 59,734 (69.7%) were smear-positive and 25,947 (30.3%) were smear negative. Of the smear-positive pulmonary TB cases 56,047 (93.8%) were new and 3,688 (6.2%) were retreatment cases.

Of the 95,745 cases of TB reported to PNLT during the period, 49,988 (52.2%) were men and 45,757 (47.8%) were women. The median age of the patients was 27 years old (interquartile range 20–41). Patients in the age groups 15–44 years represented approximately 70% of all TB cases notified.

### Expansion of diagnostic capacity.

From 2010 to 2015, a total of 32 fluorescent LED microscopes and 22 GeneXpert machines were installed throughout the high volume TB treatment centers in Haiti. During the same period, the number of diagnostic smear microscopic examinations performed at the TB health centers rose from 100,000 in 2010 to 197,100 in 2014; 134,951 were conducted in 2015. Similarly, the country went from performing 1,000 Xpert MTB/RIF tests at two TB health centers in 2011 to performing 25,000 Xpert MTB/RIF tests across 20 TB health centers in 2015.

### Overall trends in case notification and treatment success.

**Figure 2.**
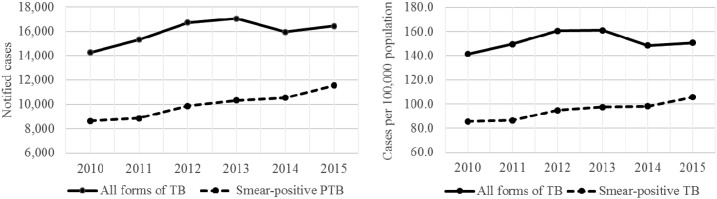
Notified cases and case notification rates for all forms of tuberculosis (TB) and smear-positive TB, Haiti.

The number of notified cases per year increased from 14,265 in 2010 to a peak of 17,040 cases in 2013, corresponding to an increase of 19.5% in the number of cases notified (Table 2, Figure 2). The increase from 2010 to 2013 represented the first time the number of notified TB cases had increased by more than 1,000 cases since 2002. From this peak, the number of cases subsequently decreased to 15,963 in 2014 and reached 16,431 in 2015. The number of smear-positive cases increased significantly from 8,624 in 2010 to 11,538 cases in 2015.

**Table 2:** Characteristics of patients with TB, Haiti, 2010–2015

Characteristics	2010	2011	2012	2013	2014	2015	Total
	*N* (%)	*N* (%)	*N* (%)	*N* (%)	*N* (%)	*N* (%)	*N* (%)
All cases	14,265	15,323	16,723	17,040	15,963	16,431	95,745
Sex							
Female	6,819 (47.8)	7,468 (48.7)	8,097 (48.4)	8,147 (47.8)	7,553 (47.3)	7,673 (46.7)	45,757 (47.8)
Male	7,446 (52.2)	7,855 (51.3)	8,626 (51.6)	8,893 (52.2)	8,410 (52.7)	8,758 (53.3)	49,988 (52.2)
Category of patient							
New	13,870 (97.2)	14,844 (96.9)	15,898 (95.1)	16,165 (94.9)	14,809 (92.8)	15,254 (92.8)	90,840 (94.9)
Retreatment	395 (2.8)	479 (3.1)	825 (4.9)	875 (5.1)	1,154 (7.2)	1,177 (7.2)	4,905 (5.1)
Age group							
0–4	782 (5.5)	834 (5.4)	1,048 (6.3)	896 (5.3)	823 (5.2)	831 (5.1)	5,214 (5.4)
5–14	789 (5.5)	952 (6.2)	978 (5.8)	1,004 (5.9)	873 (5.5)	763 (4.6)	5,359 (5.6)
15–24	3,566 (25.0)	3,865 (25.2)	4,115 (24.6)	4,112 (24.1)	3,950 (24.7)	3,992 (24.3)	23,600 (24.6)
25–34	3,912 (27.4)	4,258 (27.8)	4,647 (27.8)	4,804 (28.2)	4,528 (28.4)	4,811 (29.3)	26,960 (28.2)
35–44	2,312 (16.2)	2,406 (15.7)	2,595 (15.5)	2,717 (15.9)	2,574 (16.1)	2,635 (16.0)	15,239 (15.9)
45–54	1,459 (10.2)	1,584 (10.3)	1,707 (10.2)	1,784 (10.5)	1,549 (9.7)	1,740 (10.6)	9,823 (10.3)
55–64	853 (6.0)	842 (5.5)	977 (5.8)	990 (5.8)	969 (6.1)	960 (5.8)	5,591 (5.8)
65 and older	592 (4.2)	582 (3.8)	656 (3.9)	733 (4.3)	697 (4.4)	699 (4.3)	3,959 (4.1)
TB disease category							
All smear + TB	8,624 (60.5)	8,851 (57.8)	9,863 (59.0)	10,313 (60.5)	10,545 (66.1)	11,538 (70.2)	59,734 (62.4)
New smear + TB	8,243 (57.8)	8,399 (54.8)	9,261 (55.4)	9,678 (56.8)	9,747 (61.1)	10,718 (65.2)	56,046 (58.5)
All smear − TB	4,334 (30.4)	4,871 (31.8)	4,940 (29.5)	4,732 (27.8)	3,802 (23.8)	3,268 (19.9)	25,947 (27.1)
Extrapulmonary TB	1,307 (9.2)	1,601 (10.4)	1,920 (11.5)	1,995 (11.7)	1,616 (10.1)	1,625 (9.9)	9,064 (9.5)
HIV status							
Tested for HIV	9,517 (66.7)	11,457 (74.8)	13,523 (80.9)	14,668 (86.1)	13,968 (87.5)	14,817 (90.2)	77,950 (81.4)
HIV-positive	1,891 (19.9)	2,317 (20.2)	2,705 (20.0)	2,857 (19.5)	2,588 (18.5)	2,426 (16.4)	14,784 (19.0)
Geographic department							
Artibonite	1,550 (10.9)	1,768 (11.5)	1,830 (10.9)	1,996 (11.7)	2,099 (13.1)	2,125 (12.9)	11,368 (11.9)
Center	738 (5.2)	802 (5.2)	820 (4.9)	963 (5.7)	775 (4.9)	1,000 (6.1)	5,098 (5.3)
Grand’Anse	664 (4.7)	705 (4.6)	763 (4.6)	712 (4.2)	709 (4.4)	756 (4.6)	4,309 (4.5)
Nippes	462 (3.2)	555 (3.6)	500 (3.0)	566 (3.3)	368 (2.3)	479 (2.9)	2,930 (3.1)
North	1,864 (13.1)	1,869 (12.2)	1,918 (11.5)	1,930 (11.3)	1,618 (10.1)	1,495 (9.1)	10,694 (11.2)
North-East	429 (3.0)	404 (2.6)	459 (2.7)	467 (2.7)	588 (3.7)	645 (3.9)	2,992 (3.1)
North-West	748 (5.2)	1,057 (6.9)	1,022 (6.1)	1,009 (5.9)	957 (6.0)	979 (6.0)	5,772 (6.0)
West	5,872 (41.2)	6,492 (42.4)	7,596 (45.4)	7,492 (44.0)	6,991 (43.8)	7,086 (43.1)	41,529 (43.4)
South	1,348 (9.4)	1,140 (7.4)	1,223 (7.3)	1,298 (7.6)	1,222 (7.7)	1,164 (7.1)	7,395 (7.7)
South-East	590 (4.1)	531 (3.5)	592 (3.5)	607 (3.6)	636 (4.0)	702 (4.3)	3,658 (3.8)

HIV = human immunodeficiency virus; TB = tuberculosis.

The CNR for all forms of TB peaked at 161.6 cases/100,000 in 2013 (Figure 2) then declined to 148.6 cases/100,000 in 2014 and to 150.6 cases/100,000 in 2015, with an average CNR of 152.0 cases/100,000 (standard deviation [SD] = 7.6) over the period. The CNRs for smear-positive pulmonary TB increased steadily from 85.5 cases/100,000 in 2010 to 105.7 cases/100,000 in 2015 (Figure 2). The average CNR for smear-positive pulmonary TB was 94.7 cases/100,000 (SD = 7.7) during the period.

The treatment success rate remained stable throughout the period; it was 79% in 2010 and 80% in 2015.

### Trends in case notification by gender.

**Figure 3.**
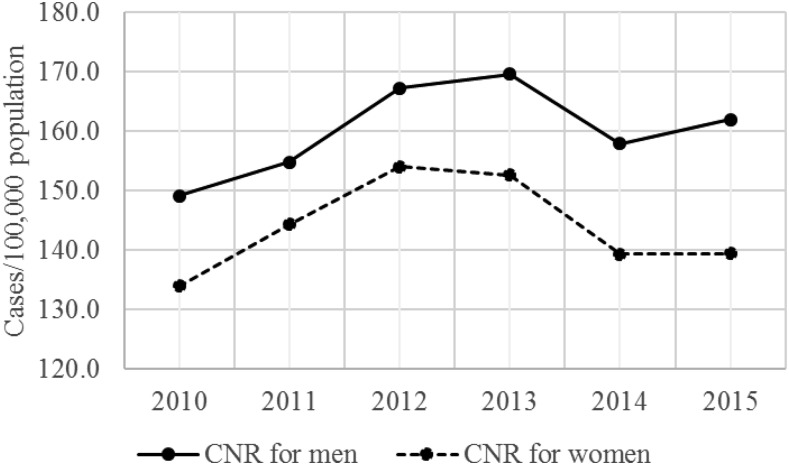
Case notification rates (CNRs) for men and women, Haiti, 2010–2015.

The number of notified cases for both men and women increased over the period of the study, reaching a peak of 8,893 cases for men and of 8,147 cases for women in 2013 (Table 2). The average number of notified cases for men was 8,331 (SD = 566), whereas that for women was 7,626 (SD = 485) (two-sample *t* test, *P* = 0.04). The average CNR for men was 160.1/100,000 (SD = 7.7), whereas that for women was 144.0/100,000 (SD = 8.0) during the period (two-sample *t* test, *P* = 0.0002) (Figure 3). The proportion of notified cases among men and women was stable over the period, with a male to female ratio of 1.1:1.

### Trends in case notification by age group.

Patients in the 15–44 age group constituted 68.7% (65,799 cases) of the TB cases notified from 2010 to 2015. For every year during the period, patients in the 0–4, 5–14, 15–24, and 25–34 age groups contributed an average of 5.4%, 5.6%, 24.7%, and 28.1%, respectively, of the total cases notified (Table 2). The number of notified cases increased over the study period in all age groups, except for the 5–14 age group, which showed a downward trend. The largest increases in notified cases were seen for the 25–34 age group, which went from 3,912 cases in 2010 to 4,811 cases in 2015 and for the more than 65 age group, which rose from 592 cases in 2010, peaked at 733 cases in 2013, then settled at 699 in 2015. Similarly, CNRs were highest for patients in the 25–34 age group (268.9 cases/100,000 in 2013), followed for those in the 35–44 (242.2/100,000 in 2013), and for those in the 15–24 age groups (191.6/100,000 in 2013) (Table 3). Conversely, CNRs were lowest for patients in the 0–14 age group (31.7–82.2 cases/100,000). There was an upward trend in CNRs in the 15–24, the 25-34, and in the over 65 age groups.

**Table 3:** Case notification per 100,000 population by age group, Haiti, 2010–2015

Age group	2010	2011	2012	2013	2014	2015
0–4	61.9	65.7	82.2	70.0	64.1	64.5
5–14	33.5	40.3	41.2	42.1	36.5	31.7
15–24	168.8	181.5	191.6	190.0	181.1	181.8
25–34	241.1	253.6	268.0	268.9	246.9	256.7
35–44	223.1	226.6	238.1	242.2	222.0	219.0
45–54	190.4	201.2	211.0	214.8	181.9	199.7
55–64	173.5	165.8	185.8	181.7	171.7	164.6
≥ 65	134.7	129.6	143.2	157.0	146.1	142.8

### Trends in case notification by geographic department.

Of Haiti’s 10 geographic departments, the West had the most cases notified for every year of the study period, reporting from 5,872 cases in 2010 and 7,086 cases in 2015, and a maximum of 7,596 (45.4%) cases reported in 2012 (Table 2). The West department, which comprises the capital Port-au-Prince, accounted for 41,529 (43.4%) of the notified cases over the 2010–2015 period. Other departments reporting relatively high numbers of cases were the Artibonite, where cases increased from 1,550 in 2010 to 2,125 in 2015 and the North, where the number of cases notified increased from 1,864 in 2010 to 1,930 in 2013, but decreased to 1,618 in 2014 and to 1,495 in 2015. The Nippes, North-East, and South-East departments had the lowest numbers of notified cases, with each department contributing 3.1%, 3.1%, and 3.8%, respectively, of the 95,745 notified cases during the period. The number of notified cases increased over the study period in the West, Artibonite, Center, North-East, North-West, South-East, and Grand’Anse departments but decreased in the Nippes, North, and South departments.

**Figure 4.**
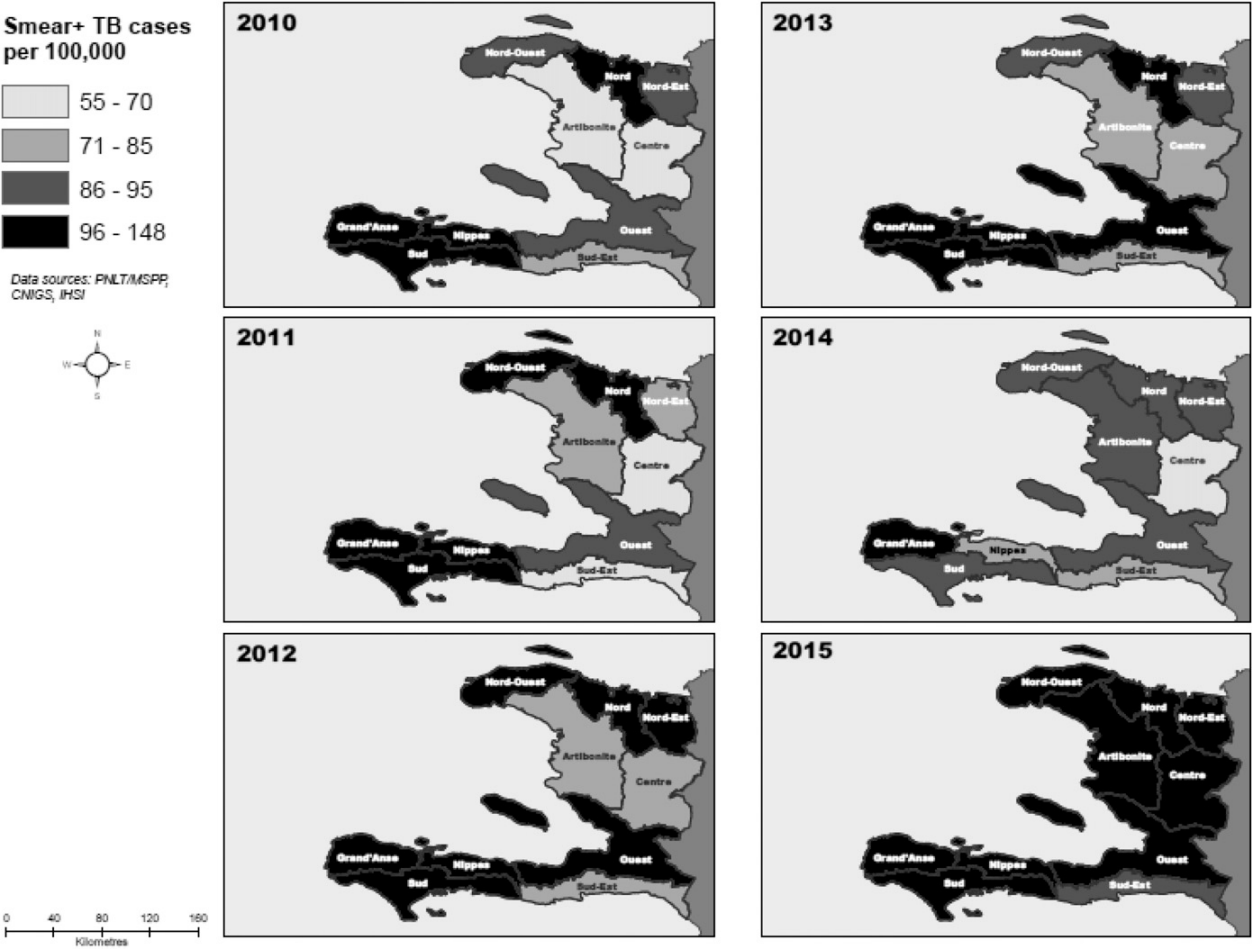
Map of departmental trends in smear + tuberculosis (TB) case notification rate, Haiti, 2010–2015.

As for CNRs, the West, Nippes, Grand’Anse, South, North, and North-West department each had more than the national average (152 cases/100,000) notified over the course of the study (Table 4). Artibonite, South-East, North-East, and Center, on the other hand, had less than 120 cases/100,000 notified during the study period. The trends in CNRs increased over the study period in the West, Artibonite, Center, North-East, North-West, South-East, and Grand’Anse departments but decreased in the Nippes, North, and South departments. The increasing trend in CNRs for smear-positive across the departments over time can be seen on the maps in Figure 4.

**Table 4:** Case notification per 100,000 population by geographic department, Haiti, 2010–2015

Department	2010	2011	2012	2013	2014	2015
Artibonite	96.1	107.8	109.8	117.9	122.1	121.7
Center	104.5	111.8	112.5	130.0	103.0	130.9
Grand’Anse	164.6	172.0	183.2	168.3	165.0	173.2
Nippes	152.7	180.5	160.1	178.3	114.2	146.3
North	184.8	182.4	184.2	182.4	150.6	137.0
North-East	106.3	98.6	110.2	110.4	136.8	147.8
North-West	123.6	171.9	163.6	159.0	148.4	149.5
West	157.4	171.2	197.2	191.4	175.8	175.5
South	190.9	158.9	167.8	175.3	162.5	152.4
South-East	97.5	86.4	94.8	95.6	98.6	107.2

### Trends in HIV testing and antiretroviral treatment coverage.

HIV testing was performed in 77,950 (81.4%) of the 95,745 patients and among those, 14,784 (19.0%) were HIV-positive (Table 2). The percentage of TB patients tested for HIV increased significantly over the period from 66.7% in 2010 to 90.2% in 2015. The percentage of patients who tested HIV-positive decreased progressively over the study period from 20% in 2010 to 16% in 2015, whereas antiretroviral treatment (ART) coverage increased from 25% in 2011 to 77% in 2015.

## DISCUSSION

This first analysis of national TB trends in Haiti showed that the number of notified cases, which had been stable at 14,000 a year since 2002, increased by approximately 20% during 2010–2015. There are several possible reasons for this increase. First, this may represent a real increase in rates of TB disease during the postearthquake period. With more than 1.5 million people homeless, thousands of TB patients initially without their medications, and an average of 200,000 people living under overcrowded conditions in camps and tent cities during the 4 years after the earthquake, the conditions were propitious for increased *Mycobacterium tuberculosis* transmission. Increased burden of TB disease has been documented in crisis-affected populations.^14^ Indeed, the number of notified smear-positive cases increased by 17% during the period and the number of pediatric TB cases, a sentinel for transmission, was also observed to be on the rise in a major Port-au-Prince clinic during 2010–2011.^6^ This indicates there may have been an increase in TB disease burden in the period following the earthquake. A second possibility is that the increase in case notification observed in our study could mean better case detection through the implementation of the activities outlined in Table 1, with a particular emphasis on cough surveillance across all facilities, active case finding activities in camps and slums, more sensitive diagnostics, and more complete reporting, at least in high burden settings, by the PNLT. Recent evaluations of TB surveillance in Haiti found the system to be functioning well and had a high level of completeness of the reporting of detected cases^10^ (Tollefson DK, unpublished report) and an analysis of the cough surveillance data showed that routine systematic screening for cough is an effective tool for increasing case finding (Richard M, personal communication). Although a prevalence survey would be potentially helpful, the decision has been to maintain focus on improving control efforts.

It is reassuring that the increase in case notification observed in our study did not have a concomitant adverse impact on treatment success, which remained stable at 79–80% during the period.

Overall CNRs were lower in children (0–14 age group), as has been documented elsewhere,^1,15–18^ highlighting the inherent challenges in the diagnosis of TB in children. The disease is paucibacillary in children and its diagnosis requires gastric aspirates, an invasive procedure that only a handful of CDTs in Haiti have the capacity to perform. As a result, most childhood TB cases in Haiti are diagnosed and treated for TB based on clinical grounds. With the expansion of GeneXpert capacity in Haiti, more children will be evaluated for TB in the coming years, but the test still requires invasive specimen collection procedures and is not yet available in many parts of the country. New and more sensitive rapid diagnostics using less invasive specimen collection are therefore needed for children suspected of having TB.^19–21^

Our analysis also shows that, while the 25–34 age group represented 14% of the general population,^13^ this age groups made up 28% of the notified cases. There was a significant increase in case notification in the group 24–35 years of age during the period. Globally, people in this age group are disproportionately affected by TB disease and this has major societal impact as people in this age group is of reproductive age and represents an active component of the workforce. The burden of disease in those 25–34, has important implications for TB control in Haiti, as they likely represent the drivers of transmission within the population. PNLT and its partners could tailor interventions and case finding efforts to focus on this age group.

The West department accounted for nearly 40% of the notified cases and showed the highest CNRs. This is not surprising, given the West accounts for nearly a third of the country’s population and for 40% of the diagnostic and treatment capacity for TB.^8,13^ Future efforts should focus on strengthening the specimen transport network in the West department to provide access to diagnostics to areas where these services are not available. Other departments with CNRs higher than the national average (152 cases/100,000), such as Grand’Anse, South, and North, should also be the focus of continued efforts to increase case finding, particularly in the more populous areas within these departments. Most active case finding activities have taken place in the Port-au-Prince metropolitan area. However, systematic screening for cough has been implemented in all departments since 2012, and to date, fluorescent LED microscopy is being expanded across high volume TB facilities and all but one department (Nippes) have had GeneXpert implemented.

The ratio of male to female TB patients in Haiti was 1.1, which is lower than the 1.7 reported for the America Region.^1^ This finding is reassuring and suggests that Haitian women may be accessing TB services at a similar level to men. It is appropriate, however, to continue advocating for TB screening services for women at all levels and types of care services, particularly in centers offering reproductive health (prenatal and obstetrical) services. This is especially true in light of a number of studies conducted elsewhere indicating that women face barriers to TB diagnosis and are less likely than men to report or show evidence of typical symptoms of pulmonary TB (cough, sputum production, and hemoptysis).^22,23^

HIV counseling and testing (HCT) among persons with TB increased by over one-third to 90% during the period. Highly encouraging, this finding indicates that TB/HIV integration has significantly improved over the last 5 years and that more TB patients are being referred for and accessing HCT services. Getting closer to 100% will require either further expansion of HIV testing or take effectively linking TB patients receiving care in facilities which do not offer testing services to others, where HCT services are available. ART coverage for TB/HIV patients is at 77%, but joint efforts must be continually made to increase the uptake of antiretroviral therapy among TB/HIV patients.

The stable treatment success rate over the period is most likely due to patients being lost to follow-up during treatment. Because of the implementation of more sensitive diagnostics (Xpert MTB/RIF and LED fluorescent microscopy), patients are increasingly being diagnosed with TB before they display more advanced symptoms of TB. As a result, individuals who receive a diagnosis of TB relatively early in the course of disease may find it difficult to accept that they have TB, a disease that still carries a high burden of stigma in Haiti. Also, because patients tend to self-transfer and because of a lack of a national health identification number to reliably track patients, it is difficult to assess reliably whether patients who are labeled as lost to follow-up are truly lost. Well aware of these challenges, PNLT has been working closely with partners to implement novel strategies to address patient retention. These include referring patients to TB centers close to their homes at the time of diagnosis, facilitating patient identification, and tracking through the use of biometric and geo-localization technology, and implementing active patient tracking after natural disasters and in areas with high loss to follow-up rates.

Going forward, PNLT will focus on increasing the treatment success rate across major TB health facilities, while targeting case finding efforts in vulnerable groups (children, detainees, and residents of slums) and in those in the 25–34 age group. Underserved communities in the urban settings will also receive particular attention through the creation of new CDTs, focused community interventions, and training.^24^

Our analysis has some limitations. The use of aggregate surveillance data in this retrospective study did not permit an analysis of patient- and health facility-level factors that may be associated with case notification. Moreover, since the last population census was conducted in 2003, it was necessary to calculate CNRs using population estimates. This approach may have led to either an under- or an overestimation of the CNRs. Finally, patients’ addresses were not available and information at the district (arrondissement) or subdistrict (commune) level was not readily available. Consequently, we had to limit our findings about CNRs to the department level and could not make inferences at lower administrative levels.

## CONCLUSIONS

Our analysis of trends in TB case notification in Haiti has provided useful information on the burden of TB in the country and will help guide future TB control efforts in Haiti. The results suggest that the investments in TB control in Haiti in the last 5 years have had an important impact leading to increasing CNRs, while treatment success remained stable. Sustaining these gains in the coming years will require that PNLT work closely with key partners and funders to focus on vulnerable populations and on underserved regions in the country.
